# DXA-Derived Total Body Fat Percentage and Serum 25(OH)D in Overweight and Obese Children: A Cross-Sectional Study

**DOI:** 10.3390/nu18142334

**Published:** 2026-07-16

**Authors:** Jolanta Świderska, Sebastian Więckowski, Michał Wilk, Wojciech Piętak, Małgorzata Wajda-Cuszlag, Anna Świercz, Maciej Jaworski, Anna Świąder-Leśniak, Agnieszka Ochocińska, Aleksandra Borowska, Marta Baszyńska-Wilk, Piotr Socha, Elżbieta Moszczyńska

**Affiliations:** 1 Department of Endocrinology and Diabetology, The Children’s Memorial Health Institute (CMHI), 04-730 Warsaw, Poland; j.swiderska@ipczd.pl (J.Ś.); w.pietak@ipczd.pl (W.P.); m.wajda-cuszlag@ipczd.pl (M.W.-C.); a.swiercz@ipczd.pl (A.Ś.); m.baszynska-wilk@ipczd.pl (M.B.-W.); e.moszczynska@ipczd.pl (E.M.); 2 Department of Gastroenterology, Hepatology, Feeding Disorders and Pediatrics, The Children’s Memorial Health Institute (CMHI), 04-730 Warsaw, Poland; s.wieckowski@ipczd.pl (S.W.); p.socha@ipczd.pl (P.S.); 3 Department of Oncology, European Health Center Otwock, 05-400 Otwock, Poland; wilk.m@onet.eu; 4 Department of Clinical Biochemistry, The Children’s Memorial Health Institute (CMHI), 04-730 Warsaw, Poland; a.ochocinska@ipczd.pl; 5 Department of Anthropometry, The Children’s Memorial Health Institute (CMHI), 04-730 Warsaw, Poland; a.swiader-lesniak@ipczd.pl; 6 Faculty of Medicine, Medical University of Gdańsk, 80-210 Gdańsk, Poland; olaborowska@onet.eu

**Keywords:** vitamin D, 25-hydroxyvitamin D, 25(OH)D, overweight, obesity, DXA, body fat percentage, BMI z-score, children, adolescents

## Abstract

**Background/Objectives:** Lower serum 25-hydroxyvitamin D [25(OH)D] is common in pediatric overweight and obesity, but it remains unclear whether direct adiposity assessment adds information beyond an age- and sex-adjusted model containing BMI z-score and season. We tested whether DXA-derived total body fat percentage is associated with 25(OH)D and whether it adds explanatory power to that model. **Methods:** We analyzed cross-sectional data from 768 children/adolescents (10–18 years) with overweight/obesity. Anthropometry was available for all; DXA data for 489. The primary analysis used complete-case DXA data (*n* = 485). Vitamin D supplementation status was not available. **Results:** Participants with and without DXA were similar in age, sex, BMI z-score, and serum 25(OH)D, but blood sampling season differed (*p* < 0.001). Adding DXA-derived body fat percentage to the age- and sex-adjusted base model increased R^2^ from 0.0498 to 0.0657 (ΔR^2^ = 0.0159). Within the complete-case DXA subgroup (*n* = 485), DXA-derived total body fat percentage was inversely associated with 25(OH)D after adjustment for BMI z-score, season, age, and sex (B = −0.265; 95% CI, −0.448 to −0.082; *p* = 0.005). BMI z-score was significant in the base model but not after adjustment for DXA fat percentage. **Conclusions:** DXA-derived total body fat percentage added explanatory value for serum 25(OH)D beyond BMI z-score and season, but the incremental gain was small (ΔR^2^ = 0.0159). The findings are consistent with BMI z-score acting as a less direct proxy for the adiposity compartment that DXA quantifies more specifically. Causal interpretation is precluded by the cross-sectional design, unknown supplementation status, and non-random DXA availability.

## 1. Introduction

Lower serum 25(OH)D levels in children and adolescents with excess body weight are well documented, and the available evidence indicates that this association is not fully captured by BMI alone. BMI does not distinguish fat mass from lean mass, which matters because vitamin D is lipophilic; its storage, distribution, and clearance depend on the size and function of the adipose compartment. Pediatric overweight and obesity also persist into adulthood and cluster with cardiometabolic, endocrine, orthopedic, and psychosocial comorbidities [1,2]. According to the World Health Organization, more than 390 million children and adolescents aged 5–19 years lived with overweight in 2022, including 160 million with obesity [3].

Low serum 25(OH)D is common in pediatric overweight and obesity. The pediatric meta-analysis by Fiamenghi and de Mello, covering more than 24,000 children and adolescents, reported a higher risk of vitamin D deficiency in obesity (RR 1.41; 95% CI, 1.26–1.59) [1]; earlier meta-analytic data across broader populations point to a stronger association [4]. Vitamin D deficiency remains a public health concern in Poland, and the current national recommendations identify overweight and obesity as risk factors that require dose adjustment [5].

Serum 25(OH)D concentration is the established marker of vitamin D status. Polish guidelines define vitamin D deficiency as <20 ng/mL (<50 nmol/L), insufficiency as 20–29 ng/mL (50–75 nmol/L), and sufficiency as ≥30 ng/mL (≥75 nmol/L). Current Central European recommendations indicate that obese children and adolescents may require vitamin D supplementation at doses approximately twice those recommended for the general pediatric population [5].

Three mechanisms could plausibly link excess adiposity to lower circulating 25(OH)D. Vitamin D is lipophilic and accumulates in adipose tissue, reducing the fraction that reaches the bloodstream [6,7]; the same hepatic output is distributed across a larger body compartment in heavier children, lowering measured serum concentrations [8]; and Roizen et al. showed in animal models that obesity reduced hepatic CYP2R1 activity, the rate-limiting 25-hydroxylase, with parallel decreases in circulating 25(OH)D [9]. A recent pediatric review reaches similar conclusions regarding volume dilution, adipose sequestration, and obesity-related changes in vitamin D hydroxylation [10]. Adipose tissue vitamin D metabolism and obesity-related inflammation have been proposed as additional contributors but are less well quantified [11,12,13]. Mendelian randomization places adiposity upstream of 25(OH)D, although cross-sectional evidence cannot establish causality [14,15].

In daily clinical practice, BMI—applied as age- and sex-specific percentiles or z-scores—remains the working tool for classifying excess body weight. Its strengths are simplicity and availability; its main limitation, well documented in pediatric populations, is that it sums fat and lean mass [16,17]. DXA, by contrast, provides direct measurement of total and regional body composition and is an established reference method in clinical research [18,19,20].

Pediatric studies have linked body composition measures, including DXA-derived adiposity and NHANES-based adiposity indices, to lower serum 25(OH)D [21,22,23,24,25,26]. Fewer studies address a narrower question: whether DXA-derived total adiposity remains associated with 25(OH)D once BMI z-score, season, age, and sex are entered into the same pediatric overweight/obesity model, and whether adding DXA-derived fat percentage adds measurable explanatory information to that age- and sex-adjusted model.

Our aim was to determine whether DXA-derived total body fat percentage was associated with serum 25(OH)D after adjustment for BMI z-score, season, age, and sex in the complete-case DXA subgroup, and whether it added explanatory information beyond that age- and sex-adjusted base model.

## 2. Materials and Methods

### 2.1. Study Design and Participants

This cross-sectional study analyzed data from 768 children and adolescents with overweight or obesity. BMI z-scores were calculated in the source anthropometric dataset using age- and sex-specific Polish OLAF reference values and the LMS method [27]. Overweight and obesity were classified using the International Obesity Task Force (IOTF) age- and sex-specific BMI cut-offs, corresponding to adult BMI thresholds of 25 kg/m^2^ and 30 kg/m^2^ at 18 years [16]. Eligible participants were aged 10 to 18 years. Pubertal stage was not systematically assessed.

We consecutively recruited participants from the Obesity Treatment Program at the Children’s Memorial Health Institute, Warsaw, Poland (Instytut “Pomnik-Centrum Zdrowia Dziecka”, IPCZD). Demographic, anthropometric, body composition, and laboratory data were extracted from clinical records covering January 2024 to October 2025. The study cohort comprised 768 children and adolescents with overweight or obesity. Serum 25(OH)D measurements were available for 755 participants, and all regression analyses involving serum 25(OH)D were performed using complete-case data. Serum 25(OH)D concentration and blood sampling season were unavailable for the same 13 participants, four of whom belonged to the DXA subgroup. DXA-derived body composition was available in 489 participants.

This was a retrospective study based solely on the analysis of existing medical records; no procedures beyond the standard clinical diagnostic work-up of children with overweight or obesity were performed. The study followed the Declaration of Helsinki and applicable data protection law (Regulation (EU) 2016/679), and the retrospective analysis was approved by the Bioethics Committee of the Children’s Memorial Health Institute (see the Institutional Review Board Statement), which found no ethical objections and confirmed that the study required no procedures beyond routine care. Consent for the clinical evaluation had been obtained from parents or legal guardians, and assent from patients aged 13 years or older, at the time of admission; the Committee approved the retrospective use of these records and did not require additional consent for the analysis.

### 2.2. Measurements

The primary exposure was DXA-derived total body fat percentage. We assessed body composition using a narrow fan beam iDXA densitometer with SmartFan technology (GE Healthcare, Madison, WI, USA) and measured bioelectrical impedance with an X-CONTACT 357S analyzer (Jawon Medical, Seoul, Republic of Korea). Both modalities followed the manufacturer’s quality control protocol.

The primary outcome was serum 25(OH)D concentration (ng/mL). The Department of Clinical Biochemistry measured total 25(OH)D on an IDS-iSYS analyzer using the IDS 25 VitDs chemiluminescence assay (Immunodiagnostic Systems Ltd., Boldon, UK). We classified vitamin D status as deficiency (<20 ng/mL), insufficiency (20–29 ng/mL), or sufficiency (≥30 ng/mL), in line with current Polish guidelines [5].

We recorded age, sex, and date of blood sampling. The season of laboratory assessment was defined by the meteorological classification and entered into the multivariable models as a categorical covariate. Vitamin D supplementation, dietary vitamin D intake, sun exposure, physical activity, and Tanner stage were not systematically available.

BMI z-score values were analyzed without truncation or winsorization, and no special handling of extreme values was applied.

BIA data were available, but this analysis was not designed as a head-to-head comparison between BIA and DXA. We chose DXA-derived total body fat percentage as the primary adiposity exposure because the central question concerned whether directly measured adiposity remains associated with 25(OH)D after adjustment for BMI z-score, season, age, and sex.

### 2.3. Statistical Analysis

Stata^®^ Software ver. 14.1 (StataCorp, College Station, TX, USA) was used for statistical analysis. Categorical variables are summarized as counts and percentages. Continuous variables are reported as mean ± standard deviation (SD) when normally distributed and as median with interquartile range (IQR) otherwise.

Associations with serum 25(OH)D were evaluated using linear regression. Regression coefficients are reported as unstandardized B with 95% confidence intervals (CIs). We set statistical significance at a two-sided *p* < 0.05. Missing data were not imputed; each model was fitted on the complete-case dataset. Stratified regression by weight status category was not performed because of the limited overweight sample size (*n* = 53; 6.9% of the cohort), which would yield unstable estimates. Because participants with obesity constituted 93.1% of the cohort, an obesity-only model would essentially reproduce the primary analysis. Descriptive characteristics of the overweight and obesity subgroups are provided in Table S2.

The primary analysis used nested models fitted in the same complete-case DXA subgroup (*n* = 485). The age- and sex-adjusted base model included BMI z-score, season of blood sampling, age, and sex. The DXA model additionally included DXA-derived total body fat percentage. Incremental explanatory information was described using R^2^, adjusted R^2^, and ΔR^2^. We did not perform an a priori power calculation; sample size was determined by data availability during the study period.

## 3. Results

**Figure 1:**
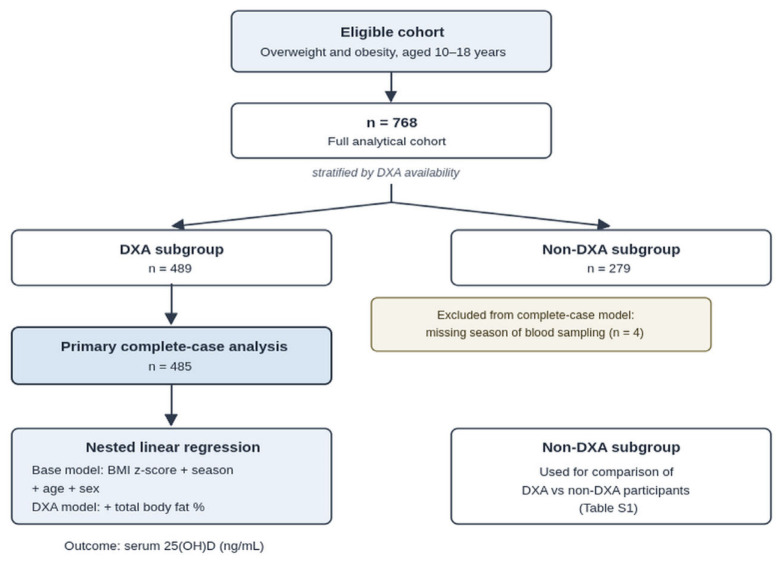
Participant flow. From an eligible cohort of children and adolescents with overweight or obesity aged 10 to 18 years (*n* = 768), DXA-derived body composition was available for 489 participants. The primary complete-case DXA analysis (*n* = 485) excluded four participants with missing season of blood sampling. The non-DXA subgroup (*n* = 279) was used for between-group comparison (Table S1) and was not included in the primary nested-model analysis.

The full cohort included 768 children and adolescents with overweight or obesity. DXA-derived body composition was available for 489 participants; the primary complete-case DXA analysis included 485 participants. Serum 25(OH)D measurements were available for 755 of the 768 participants. Analyses involving serum 25(OH)D were performed using complete-case data. The participant flow is shown in Figure 1. Baseline characteristics of the full cohort are shown in Table 1.

Participants with and without DXA assessment were similar in age, sex distribution, BMI z-score, and serum 25(OH)D. The seasonal distribution of blood sampling, however, differed between DXA and non-DXA participants (*p* < 0.001), with autumn over-represented in the DXA subgroup and spring over-represented in the non-DXA subgroup (Table S1). We retained season as a covariate in the primary DXA model.

Within the DXA subgroup (*n* = 489), the median age was 13.8 years (IQR, 12.2–15.7), 45.6% were female, the median BMI z-score was 2.36 (IQR, 2.02–2.69), the median serum 25(OH)D was 23.3 ng/mL (IQR, 18.7–28.9), and the median DXA-derived total body fat percentage was 46.7% (IQR, 42.9–50.0). The DXA-derived total body fat percentage by sex and by vitamin D status is reported in Table S3. The DXA subgroup was broadly comparable with the non-DXA subgroup for measured baseline variables, including age, sex, BMI z-score, and serum 25(OH)D; however, the seasonal distribution of blood sampling differed between groups (Table S1).

### Incremental Value of DXA-Derived Total Body Fat Percentage

Median DXA-derived total body fat percentage was 46.7% (IQR 42.9–50.0), with a mean of 46.1 ± 5.6% and a range of 30.2–62.6%. In the complete-case DXA subgroup (*n* = 485), BMI z-score was inversely associated with serum 25(OH)D in the age- and sex-adjusted base model (B = −2.98; 95% CI, −4.65 to −1.31; *p* < 0.001). Winter and spring were also associated with lower 25(OH)D than summer.

Adding DXA-derived total body fat percentage to the age- and sex-adjusted base model produced an inverse association with 25(OH)D (B = −0.265; 95% CI, −0.448 to −0.082; *p* = 0.005). In the same model, BMI z-score lost statistical significance (B = −0.98; 95% CI, −3.14 to 1.17; *p* = 0.370). The model R^2^ rose from 0.0498 to 0.0657, corresponding to ΔR^2^ = 0.0159 (Table 2).

## 4. Discussion

In the complete-case DXA subgroup, DXA-derived total body fat percentage added explanatory information on serum 25(OH)D beyond the age- and sex-adjusted base model containing BMI z-score and season. BMI z-score lost statistical significance when both adiposity variables entered the same model, whereas DXA-derived fat percentage remained inversely associated with 25(OH)D after adjustment for BMI z-score, season, age, and sex. Adding DXA-derived fat percentage increased R^2^ from 0.0498 to 0.0657 (ΔR^2^ = 0.0159). This pattern is consistent with BMI z-score serving as a less direct proxy for the adiposity compartment that DXA quantifies more specifically. These findings are consistent with DXA-derived total body fat percentage capturing the adiposity component more directly than BMI z-score in this cohort.

The size of this incremental gain should be interpreted realistically. A ΔR^2^ of 0.0159 represents a small change in explained variance, and no causal conclusion follows from it. To put the coefficient in context, a 15% difference in DXA fat corresponds to ~4.0 ng/mL lower 25(OH)D. The main finding of the present study is that, within the same participants, DXA-derived fat percentage retained explanatory information beyond BMI z-score. We used total body fat percentage as the single DXA adiposity index because the research question concerned whole-body adiposity rather than fat distribution. Percentage of body fat expresses adiposity independently of body size and corresponds directly to the exposure examined here, whereas absolute fat mass and lean mass scale with height and pubertal stage and would require additional height- and maturation-based standardization for comparison across a 10-to-18-year range. Regional compartments describe fat distribution, which fell outside the predefined objectives.

Our results are consistent with, and more specific than, earlier pediatric evidence on adiposity and vitamin D status. The pediatric meta-analysis by Fiamenghi and de Mello reported an increased risk of vitamin D deficiency among children and adolescents with obesity [1]. In NHANES 2005–2006, Moore and Liu found inverse associations between serum 25(OH)D and several adiposity measures in children aged 6–18 years; in an 8–18-year subgroup, DXA-derived body fat, fat mass index, and percent body fat were also negatively associated with serum 25(OH)D [25]. Wang et al., using NHANES 2011–2018 adolescent data, linked vitamin D status to DXA-derived body composition measures, including visceral adipose tissue area, appendicular lean mass index, and bone mineral density [26]. Our analysis sharpens these observations by asking a narrower question: whether DXA-derived total body fat percentage adds explanatory information beyond an age- and sex-adjusted model containing BMI z-score and season in the same complete-case pediatric overweight/obesity sample.

The biological rationale is strongest when adiposity is considered directly rather than through BMI. Cholecalciferol and vitamin D metabolites are lipophilic and, once stored in the adipose pool, re-enter the circulation slowly [6]. The same hepatic output, distributed across a larger body compartment, results in lower measured serum concentrations [8], and obesity-related reductions in CYP2R1-mediated 25-hydroxylation further lower 25(OH)D [9]. Adipose tissue itself expresses vitamin D-metabolizing enzymes (CYP27B1 and CYP24A1), and obesity-related low-grade inflammation can interfere with VDR-mediated transcription [13,28,29]; experimental data from vitamin D receptor knockout models confirm the centrality of VDR signaling for calcium, bone, and immune homeostasis [30]. None of these mechanisms scales cleanly with the BMI z-score because BMI mixes fat and lean mass in proportions that vary with sex and pubertal stage; they scale with fat mass, which is what DXA measures.

The adipose context runs in both directions. Vitamin D signaling has been linked experimentally to adipocyte differentiation via PPAR-γ, to lipolysis and insulin sensitivity, and to attenuated pro-inflammatory cytokine release in adipose tissue [31,32]. None of these associations were tested in our cohort; the point here is that DXA-derived fat percentage reflects the compartment in which these processes occur, whereas the BMI z-score does not.

Circulating vitamin D derives mainly from cutaneous synthesis after UVB exposure, with only a minor contribution from diet [4,28,33]. In Poland, cutaneous synthesis falls markedly in autumn and winter, which is consistent with the seasonal differences in 25(OH)D observed here. Dietary vitamin D intake, sun exposure, and physical activity were not measured in the present study, so their contribution cannot be quantified; residual confounding from these factors cannot be excluded.

Season remained relevant in the model. In the DXA model, winter and spring were associated with lower 25(OH)D than summer, with effect sizes (winter B = −3.15; spring B = −2.99) close to those in the base model. Participants with and without DXA measurements were comparable with respect to age, sex, BMI z-score, and serum 25(OH)D concentrations. However, the distribution of blood sampling season differed between groups. To minimize potential confounding arising from this imbalance, season was included as a covariate in all multivariable analyses, but residual season-related selection bias cannot be excluded. The pattern is consistent with Polish pediatric data from central Poland, where 25(OH)D shows marked seasonal variation and deficiency clusters in periods of lower UVB exposure [34].

The recent analysis by Jakubowska-Pietkiewicz et al. and the present study ask different questions [35]. Their study examined whether DXA and BIA yield similar physiological inferences when modeling adiposity–25(OH)D and vitamin D–endocrine/metabolic associations in children with established obesity. Our analysis does not test whether DXA outperforms BIA; it asks whether DXA-derived total body fat percentage adds explanatory information beyond an age- and sex-adjusted model containing BMI z-score and season in a broader clinical cohort that includes both overweight and obesity. Vitamin D deficiency in our overweight/obesity cohort was 31.0%, lower than the 62.4% reported by Jakubowska-Pietkiewicz et al. Direct comparison is limited by differences in seasonal distribution, supplementation status, cohort composition, and assay methodology.

Vitamin D supplementation status was unknown in our cohort. Because supplementation directly affects serum 25(OH)D concentrations, this is an important unmeasured source of confounding. Many participants had 25(OH)D levels below the recommended threshold, but we could not assess the adequacy of supplementation, adherence, dose, or duration. Therefore, the present findings should not be interpreted as evidence that current supplementation was ineffective. Polish guidelines explicitly recommend higher vitamin D doses for children and adolescents with overweight or obesity than for normal-weight peers, typically up to two-fold, to reach 25(OH)D concentrations within the recommended range [5]. Recent Endocrine Society recommendations address empiric vitamin D supplementation in children and adolescents but do not provide DXA-based dose calibration for pediatric obesity. Current clinical guidelines do not recommend routine 25(OH)D screening, including for individuals with obesity [36]. Randomized trials and meta-analyses show that supplementation can raise 25(OH)D, but its effects on adiposity or metabolic outcomes are inconsistent and do not replace standard obesity management [37,38,39].

Our finding does not mean that every child with overweight or obesity should undergo DXA for vitamin D assessment. It indicates that, where DXA data are already available in a research or specialist care setting, total body fat percentage is a more direct measure of adiposity than BMI z-score and describes the adiposity–25(OH)D relationship more closely. DXA still has clear constraints—cost, access, specialized equipment, and low-dose radiation. Its most defensible role in this context is to characterize adiposity when precise body composition assessment is clinically or scientifically justified. The present data do not support DXA-based vitamin D dose calibration because supplementation dose, adherence, and treatment response were unavailable. They suggest, however, that direct adiposity assessment carries vitamin D-relevant information that weight-for-height indices alone do not retain.

The study has clear limitations. The cross-sectional design rules out temporal or causal claims. We did not have systematic data on vitamin D supplementation, diet, sun exposure, physical activity, or pubertal stage, all of which can affect 25(OH)D and act as residual confounders. DXA was available only in a subgroup, and selection for DXA was non-random; although age, sex, BMI z-score, and 25(OH)D were similar between DXA and non-DXA participants, the seasonal distribution of blood sampling differed significantly, so season-related selection bias cannot be excluded even after model adjustment. The cohort was recruited from a tertiary obesity program, which limits generalizability to community pediatric populations. DXA fat mass, lean mass, and regional adiposity were not analyzed, so compartment- and distribution-specific associations with 25(OH)D cannot be addressed by these data. Strengths include DXA-based assessment of adiposity in 489 children and adolescents with overweight or obesity, the use of complete-case nested models within the same DXA subgroup, and simultaneous adjustment for BMI z-score, season, age, and sex in the primary model.

## 5. Conclusions

In the complete-case DXA subgroup (*n* = 485) of a cohort of 768 children and adolescents with overweight or obesity, DXA-derived total body fat percentage added modest explanatory information on serum 25(OH)D beyond an age- and sex-adjusted base model containing BMI z-score and season (ΔR^2^ = 0.0159) and remained inversely associated with 25(OH)D after adjustment for BMI z-score, season, age, and sex, whereas BMI z-score lost statistical significance once both adiposity variables were entered. These findings are consistent with BMI z-score serving as a less direct proxy for adiposity than DXA-derived total body fat percentage in this cohort. Because the design was cross-sectional and supplementation, dietary, UV exposure, and pubertal stage data were unavailable, causal interpretation is not possible, and DXA-derived total body fat percentage should not be used as a basis for routine vitamin D screening or dosing; longitudinal and interventional studies with these variables recorded would be the most informative next step. DXA-derived total body fat percentage may help characterize the adiposity–25(OH)D association in specialist or research settings where DXA has already been obtained.

## Figures and Tables

**Table 1:** Characteristics of the study population.

Variable	Study Cohort (*n* = 768)
Age, years—mean ± SD (range)	14.0 ± 2.2 (10.0–18.0)
Age, years—median (IQR)	13.9 (12.2–15.8)
Girls, *n* (%)	359 (46.7)
Boys, *n* (%)	409 (53.3)
DXA-derived total body fat (%), median (IQR), *n* = 489	46.7 (42.9–50.0)
DXA-derived total body fat (%), mean ± SD (range), *n* = 489	46.1 ± 5.6 (30.2–62.6)
BMI z-score—mean ± SD	2.4 ± 0.6
BMI z-score—median (IQR)	2.34 (2.0–2.7)
Serum 25(OH)D, ng/mL—mean ± SD	23.8 ± 8.4
Serum 25(OH)D, ng/mL—median (IQR)	23.1 (18.2–28.5)
Vitamin D deficiency, *n* (%)	238 (31.0)
Vitamin D insufficiency, *n* (%)	363 (47.3)
Vitamin D sufficiency, *n* (%)	154 (20.1)
Missing 25(OH)D status, *n* (%)	13 (1.6)
Spring sampling, *n* (%)	294 (38.3)
Summer sampling, *n* (%)	111 (14.5)
Autumn sampling, *n* (%)	173 (22.5)
Winter sampling, *n* (%)	177 (23.0)
Missing season data, *n* (%)	13 (1.6)
Weight status, *n* (%)	
Overweight	53 (6.9)
Obesity	715 (93.1)

Data are presented as mean ± SD, median (interquartile range, IQR), or number (percentage), as appropriate. Vitamin D status was defined as deficiency (<20 ng/mL), insufficiency (20–29 ng/mL), and sufficiency (≥30 ng/mL). DXA-derived total body fat percentage was available only for the DXA subgroup (*n* = 489). Percentages for vitamin D status and season are calculated against the full cohort denominator; missing values are shown explicitly.

**Table 2:** Incremental value of DXA-derived total body fat percentage in explaining serum 25(OH)D concentration in the complete-case DXA subgroup.

Variable	Base B	Base 95% CI	Base p	DXA B	DXA 95% CI	DXA p
DXA—total body fat (%)	—	—	—	−0.265	−0.448 to −0.082	0.005
BMI z-score	−2.98	−4.65 to −1.31	<0.001	−0.98	−3.14 to 1.17	0.370
Age (years)	0.07	−0.31 to 0.45	0.730	−0.20	−0.62 to 0.22	0.345
Sex (female vs. male)	−0.36	−1.90 to 1.18	0.646	0.18	−1.40 to 1.75	0.826
**Season (reference: summer)**						
Autumn	−1.52	−3.86 to 0.83	0.204	−1.67	−4.00 to 0.66	0.160
Winter	−2.92	−5.42 to −0.42	0.022	−3.15	−5.64 to −0.66	0.013
Spring	−2.94	−5.34 to −0.54	0.016	−2.99	−5.38 to −0.62	0.014
N	**485**			**485**		
R^2^	0.0498			0.0657		
Adjusted R^2^	0.0379			0.0520		
ΔR^2^				0.0159		

Both models were fitted in the DXA complete-case subgroup (n = 485). The base model included BMI z-score, season, age, and sex. The DXA model additionally included DXA-derived total body fat percentage. B coefficients represent differences in serum 25(OH)D concentration (ng/mL). Abbreviations: CI, confidence interval; DXA, dual-energy X-ray absorptiometry; 25(OH)D, 25-hydroxyvitamin D.

## Data Availability

The data presented in this study are available upon request from the corresponding author. The data are not publicly available due to privacy and ethical restrictions related to clinical pediatric information.

## Supplementary Materials

The following supporting information can be downloaded at: https://www.mdpi.com/article/10.3390/nu18142334/s1. Table S1: comparison of participants with and without DXA assessment; Table S2: comparison of the overweight and obesity subgroups (full cohort, *n* = 768); Table S3: DXA-derived total body fat percentage in the DXA subgroup (*n* = 489), by sex and by vitamin D status.

## References

[1] Fiamenghi V.I., de Mello E.D. (2021). Vitamin D deficiency in children and adolescents with obesity: A meta-analysis. J. Pediatr..

[2] Mazur A., Zachurzok A., Baran J., Dereń K., Łuszczki E., Weres A., Wyszyńska J., Dylczyk J., Szczudlik E., Drożdż D. (2022). Childhood obesity: Position statement of Polish societies. Nutrients.

[3] World Health Organization (2024). Obesity and Overweight. https://www.who.int/news-room/fact-sheets/detail/obesity-and-overweight.

[4] Pereira-Santos M., Costa P.R.F., Assis A.M.O., Santos C.A.S.T., Santos D.B. (2015). Obesity and vitamin D deficiency: A systematic review and meta-analysis. Obes. Rev..

[5] Płudowski P., Kos-Kudła B., Walczak M., Fal A., Zozulińska-Ziółkiewicz D., Sieroszewski P., Peregud-Pogorzelski J., Lauterbach R., Targowski T., Lewiński A. (2023). Guidelines for preventing and treating vitamin D deficiency: A 2023 update in Poland. Nutrients.

[6] Wortsman J., Matsuoka L.Y., Chen T.C., Lu Z., Holick M.F. (2000). Decreased bioavailability of vitamin D in obesity. Am. J. Clin. Nutr..

[7] Lee M.-J. (2025). Vitamin D enhancement of adipose biology: Implications on obesity-associated cardiometabolic diseases. Nutrients.

[8] Drincic A.T., Armas L.A.G., Van Diest E.E., Heaney R.P. (2012). Volumetric dilution explains the low vitamin D status of obesity. Obesity.

[9] Roizen J.D., Long C., Casella A., O’Lear L., Caplan I., Lai M., Sasson I., Singh R., Makowski A.J., Simmons R. (2019). Obesity decreases hepatic 25-hydroxylase activity causing low vitamin D status. J. Bone Miner. Res..

[10] Zhou X., Chen Y., Xu J., Li X., Wu Y., Xu L. (2026). The interplay between childhood obesity and vitamin D deficiency: Mechanisms and implications. Front. Pediatr..

[11] Walsh J.S., Bowles S., Evans A.L. (2017). Vitamin D in obesity. Curr. Opin. Endocrinol. Diabetes Obes..

[12] Earthman C.P., Beckman L.M., Masodkar K., Sibley S.D. (2012). The link between obesity and low circulating 25-hydroxyvitamin D concentrations: Considerations and implications. Int. J. Obes..

[13] Karampela I., Sakelliou A., Vallianou N., Christodoulatos G.S., Magkos F., Dalamaga M. (2021). Vitamin D and obesity: Current evidence and controversies. Curr. Obes. Rep..

[14] Vimaleswaran K.S., Berry D.J., Lu C., Tikkanen E., Pilz S., Hiraki L.T., Cooper J.D., Dastani Z., Li R., Houston D.K. (2013). Causal relationship between obesity and vitamin D status: Bi-directional Mendelian randomization analysis of multiple cohorts. PLoS Med..

[15] Luo X., Luo J., Du J., Sun X., He K., Zhu Y., Lu D., Gu H. (2025). Association between childhood obesity and vitamin D: A Mendelian randomization study. Pediatr. Int..

[16] Cole T.J., Lobstein T. (2012). Extended International (IOTF) body mass index cut-offs for thinness, overweight and obesity. Pediatr. Obes..

[17] Freedman D.S., Wang J., Thornton J.C., Mei Z., Sopher A.B., Pierson R.N., Dietz W.H., Horlick M. (2009). Classification of body fatness by body mass index-for-age categories among children. Arch. Pediatr. Adolesc. Med..

[18] Earthman C.P. (2015). Body composition tools for assessment of adult malnutrition at the bedside. J. Parenter. Enter. Nutr..

[19] Borga M., West J., Bell J.D., Harvey N.C., Romu T., Heymsfield S.B., Leinhard O.D. (2018). Advanced body composition assessment: From body mass index to body composition profiling. J. Investig. Med..

[20] Kelly T.L., Wilson K.E., Heymsfield S.B. (2009). Dual energy X-ray absorptiometry body composition reference values from NHANES. PLoS ONE.

[21] Kouda K., Nakamura H., Fujita Y., Ohara K., Iki M. (2013). Vitamin D status and body fat measured by DXA in Japanese children. Nutrition.

[22] Fu Z., Xu C., Shu Y., Xie Z., Lu C., Mo X. (2020). Serum 25-hydroxyvitamin D and obesity in children and adolescents. Public Health Nutr..

[23] Lenders C.M., Feldman H.A., von Scheven E., Merewood A., Sweeney C., Wilson D.M., Lee P.D., Abrams S.H., Gitelman S.E., Wertz M.S. (2009). Relation of body fat indexes to vitamin D status and deficiency among obese adolescents. Am. J. Clin. Nutr..

[24] Kasvis P., Cohen T.R., Loiselle S., Hazell T.J., Vanstone C.A., Weiler H.A. (2022). Body composition and vitamin D status in healthy children. Nutrients.

[25] Moore C.E., Liu Y. (2016). Low serum 25-hydroxyvitamin D concentrations are associated with total adiposity of children in the United States: National Health and Nutrition Examination Survey 2005 to 2006. Nutr. Res..

[26] Wang B., Gao S., Zhu Z. (2025). Vitamin D deficiency and adverse body composition in adolescents. J. Orthop. Surg. Res..

[27] Kułaga Z., Litwin M., Tkaczyk M., Palczewska I., Zajączkowska M., Zwolińska D., Krynicki T., Wasilewska A., Moczulska A., Morawiec-Knysak A. (2011). Polish 2010 growth references for school-aged children and adolescents. Eur. J. Pediatr..

[28] Bikle D., Christakos S. (2020). New aspects of vitamin D metabolism and action-addressing the skin as source and target. Nat. Rev. Endocrinol..

[29] Christakos S., Dhawan P., Verstuyf A., Verlinden L., Carmeliet G. (2016). Vitamin D: Metabolism, molecular mechanism of action, and pleiotropic effects. Physiol. Rev..

[30] Bouillon R., Bikle D., Kovacs C.S., Vieth R., White J.H. (2022). Vitamin D and human health: Lessons from vitamin D receptor null mice. Endocr. Rev..

[31] Nimitphong H., Park E., Lee M.J. (2020). Vitamin D regulation of adipogenesis and adipose tissue functions. Nutr. Res. Pract..

[32] Szymczak-Pajor I., Drzewoski J., Śliwińska A. (2020). The molecular mechanisms by which vitamin D prevents insulin resistance and associated disorders. Int. J. Mol. Sci..

[33] Benedik E. (2022). Sources of vitamin D for humans. Int. J. Vitam. Nutr. Res..

[34] Smyczyńska J., Smyczyńska U., Stawerska R., Domagalska-Nalewajek H., Lewiński A., Hilczer M. (2019). Seasonality of vitamin D concentrations and the incidence of vitamin D deficiency in children and adolescents from central Poland. Pediatr. Endocrinol. Diabetes Metab..

[35] Jakubowska-Pietkiewicz E., Chrzanowski J., Woźniak E. (2026). Densitometry versus bioimpedance for modeling vitamin D-endocrine and metabolic associations in pediatric obesity: A cross-sectional parallel-modality analysis. Nutrients.

[36] Demay M.B., Pittas A.G., Bikle D.D., Diab D.L., E Kiely M., Lazaretti-Castro M., Lips P., Mitchell D.M., Murad M.H., Powers S. (2024). Vitamin D for the prevention of disease. J. Clin. Endocrinol. Metab..

[37] Ganmaa D., Bromage S., Khudyakov P., Erdenenbaatar S., Delgererekh B., Martineau A.R. (2023). Influence of vitamin D supplementation on growth, body composition, and pubertal development. JAMA Pediatr..

[38] Gou H., Wang Y., Liu Y., Peng C., He W., Sun X. (2023). Efficacy of vitamin D supplementation on child and adolescent overweight/obesity: A systematic review and meta-analysis of randomized controlled trials. Eur. J. Pediatr..

[39] Rajakumar K., Moore C.G., Khalid A.T., Vallejo A.N., Virji M.A., Holick M.F., Greenspan S.L., Arslanian S., Reis S.E. (2020). Effect of vitamin D3 supplementation on vascular and metabolic health of vitamin D-deficient overweight and obese children: A randomized clinical trial. Am. J. Clin. Nutr..

