# Supplementary material for: DXA-Derived Total Body Fat Percentage and Serum 25(OH)D in Overweight and Obese Children: A Cross-Sectional Study
Source: Nutrients. 2026 Jul 16;18(14):2334. doi: 10.3390/nu18142334 (PMC13415124; doi:10.3390/nu18142334)
Supplement: Supplementary file 1 [file nutrients-18-02334-s001.zip › nutrients-4406264-supplementary.pdf]

Table S1. Comparison of participants with and without DXA assessment.

Variable	DXA Subgroup (*n* = 489)	Non-DXA Subgroup (*n* = 279)	p-Value
Age, years — median (IQR)	13.8 (12.2–15.7)	14.0 (12.2–16.1)	0.178
Female sex, *n* (%)	223 (45.6%)	136 (48.8%)	0.401
BMI z-score — median (IQR)	2.36 (2.02–2.69)	2.31 (1.97–2.72)	0.225
Serum 25(OH)D, ng/mL — median (IQR)	23.3 (18.7–28.9)	22.8 (17.5–28.1)	0.165
**Blood sampling season, *n* (%)**			<0.001
Spring	139 (28.7%)	155 (57.4%)	
Summer	70 (14.4%)	41 (15.2%)	
Autumn	162 (33.4%)	11 (4.1%)	
Winter	114 (23.5%)	63 (23.3%)	

Values are presented as median (IQR) or number (percentage). DXA = dual-energy X-ray absorptiometry; 25(OH)D = 25-hydroxyvitamin D. Percentages for blood sampling season were calculated using non-missing data (missing season data: DXA subgroup = 4; non-DXA subgroup = 9).

Table S2. Comparison of the overweight and obesity subgroups (full cohort, *n* = 768).

Variable	Overweight (*n* = 53)	Obesity (*n* = 715)	p
Age, years — median (IQR)	12.7 (11.6–13.9)	14.0 (12.2–15.8)	<0.001
Female sex, *n* (%)	14 (26.4)	345 (48.3)	0.002
BMI z-score — median (IQR)	1.48 (1.37–1.58)	2.38 (2.08–2.74)	<0.001
Serum 25(OH)D, ng/mL — median (IQR)*	27.2 (23.8–33.1) *n* = 53	22.8 (17.7–28.0) *n* = 702	<0.001
DXA total body fat, % — median (IQR)**	39.6 (34.5–43.7) *n* = 27	46.9 (43.5–50.0) *n* = 462	<0.001
Blood sampling season, *n* (%)			
Spring	21 (39.6)	273 (38.9)	0.135
Summer	13 (24.5)	98 (14.0)	
Autumn	11 (20.8)	162 (23.1)	
Winter	8 (15.1)	169 (24.1)	

Overweight and obesity are defined by IOTF BMI cut-offs; the groups differ by design on BMI-related measures, and the reported p-values are descriptive. DXA-derived total body fat percentage was available only in the DXA subgroup, so the analysed n is shown per group.

Table S3. DXA-derived total body fat percentage in the DXA subgroup (*n* = 489), by sex and by vitamin D status.

Stratum	*n*	Median (IQR)	Mean ± SD	Range
Girls	223	47.8 (45.2–50.8)	48.0 ± 4.2	35.2–62.6
Boys	266	45.4 (40.5–49.4)	44.6 ± 6.1	30.2–59.5
25(OH)D deficiency (< 20 ng/mL)	146	47.7 (44.7–50.7)	47.5 ± 4.9	33.1–60.1
25(OH)D insufficiency (20–29 ng/mL)	233	46.4 (42.6–49.9)	45.8 ± 5.7	30.2–59.5
25(OH)D sufficiency (≥ 30 ng/mL)	106	45.0 (40.8–49.1)	45.0 ± 6.0	30.6–62.6

Vitamin D status could not be classified for four DXA-subgroup participants with missing serum 25(OH)D, so the by-status rows include 485 participants. DXA, dual-energy X-ray absorptiometry; IQR, interquartile range; SD, standard deviation.
